# Delayed Post-operative Spinal Epidural Haematoma after Posterior Spinal Surgery: Report of Two Cases

**DOI:** 10.5704/MOJ.2011.027

**Published:** 2020-11

**Authors:** WH Chung, RL Tan, CK Chiu, MK Kwan, CYW Chan

**Affiliations:** Department of Orthopaedic Surgery, University of Malaya, Kuala Lumpur, Malaysia

**Keywords:** delayed post-operative spinal epidural haematoma, spinal surgery, complication, neurological deficit

## Abstract

Delayed post-operative spinal epidural haematoma (DPSEH) is diagnosed when the onset of symptoms is more than three days from the index surgery. DPSEH is a rare but serious complication of spinal surgery. Missed diagnosis will result in irreversible neurological deficit which may lead to permanent disabilities. We report two cases of DPSEH who presented with worsening neurological deficit four days after the index surgery. Magnetic resonance imaging (MRI) showed the presence of an epidural haematoma compressing the spinal cord. Surgical evacuation of haematoma were performed for both patients. Both patients experienced neurological improvement. Surgeons should have high index of suspicion to identify delayed onset of spinal epidural haematoma (SEH) and timely intervention should be taken to avoid irreversible neurological damage.

## Introduction

Post-operative spinal epidural haematoma (PSEH) is a rare complication of spinal surgery^1^. Prompt recognition is essential to prevent irreversible neurological deficits. It was estimated that 33% of patients had PSEH based on magnetic resonance imaging (MRI) findings, but only 0.1-3% were symptomatic and required surgical evacuation^2^. Delayed PSEH (DPSEH) is diagnosed when the onset of symptoms was more than three days from the index surgery^3^. Uribe *et al*, in a review of 4,018 patients, reported an incidence of 0.17%^3^. Patients could present with worsening surgical site pain or new onset neurological deficit. It is difficult to ascertain whether PSEH was the cause of pain and neurological deficit because the post-operative imaging will usually demonstrate presence of haematoma^3^. Due to its rarity, surgeons might not suspect DPSEH and this may lead to irreversible neurological deficits. Therefore, it is important to have a high index of suspicion to diagnose DPSEH. In this series, we report two cases of DPSEH, its clinical presentation and their treatment outcome.

## Case Report

### Case 1

**Fig. 1:**
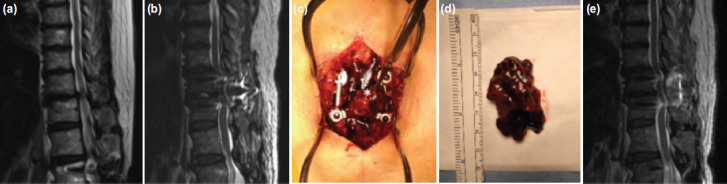
Case 1; (a) MRI showing T11/T12 stenosis due to degenerative disc disease and facet arthropathy. (b) MRI showing spinal epidural haematoma with spinal cord compression; (c,d) intra-operative photo showing spinal epidural haematoma (white asterisk in c); and (e) MRI after evacuation of haematoma showing complete decompression of the spinal cord.

A 76-year-old female complained of unsteady gait and urinary incontinence for a duration of one year. She was able to walk with a walking stick. She had a history of L2-S1 instrumented fusion with L2-L5 laminectomy for degenerative spine disease nine years ago. Post-operatively, her neurology was normal. She was otherwise healthy. Neurological examination revealed full motor power, but presence of knee hyperreflexia and reduced sensation below L1 on the left side. MRI showed T11/T12 stenosis due to degenerative disc disease and facet arthropathy (Fig. 1a). Blood investigations were normal. She underwent posterior decompression, instrumentation and posterolateral fusion of T11/T12 level. The surgery was uneventful. There was no excessive intra-operative bleeding. One Radivac drain was inserted. The drain was removed at post-operative day 1. Post-operative neurological examination was normal. She was well until post-operative day 4 where she complained of worsening lower limb weakness (worse on right side) (Medical Research Council (MRC) power grade 3 to 4 on right side; grade 4 to 5 on left side). The weakness worsened when she was lying in supine position but improved in lateral position. An urgent MRI was done which revealed SEH at the surgical site (T11/T12 level) (Fig. 1b). A diagnosis of DPSEH was made and patient was scheduled for surgical evacuation of the haematoma (Fig. 1c and d). Postoperatively, patient’s motor power improved (increased one grade). Post-operative MRI demonstrated complete decompression of the spinal cord (Fig. 1e). She underwent intensive inpatient rehabilitation and six weeks later, she was able to walk with a walking frame. At two-year follow-up, she was well with similar neurological examination findings and walked with a quadripod.

### Case 2

**Fig. 2:**
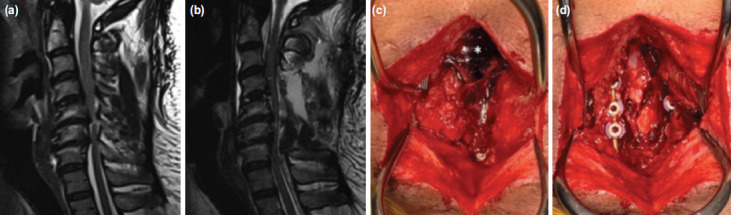
Case 2; (a) MRI showing C4/5, C5/6 and C6/7 spinal stenosis with spinal cord oedema. (b) MRI showing spinal epidural haematoma with spinal cord compression; and (c) intra-operative photo showing spinal epidural haematoma (white asterisk) and (d) complete decompression of the spinal cord after evacuation of haematoma.

A 53-year-old gentleman presented with bilateral upper limb numbness, gait instability and urinary incontinence for four months. He was able to walk without any assistive device. He had diabetes mellitus (well controlled) and coronary artery disease. He was taking aspirin 80 mg daily which was withheld five days prior to surgery. MRI showed a C4/5, C5/6 and C6/7 spinal stenosis with spinal cord oedema (Fig. 2a). He was diagnosed of having cervical spondylotic myelopathy (Japanese Orthopaedic Association score of 14). Pre-operative laboratory results were normal. He underwent total laminectomy from C4 to C6, partial laminectomy of C7 and lateral mass fusion from C4 to C6 level. Surgery was uneventful. There was no excessive intra-operative bleeding. One Radivac drain was inserted. The drain was removed at post-operative day 2. Post-operatively, his symptoms improved. He was well until at post-operative day 4 when he complained of severe neck pain with weakness of right upper limb and numbness of both thighs. An urgent MRI was performed which showed SEH at the surgical area (Fig. 2b). Surgical evacuation of haematoma was performed (Fig. 2c and d). His neurological status returned to pre-operative status within two weeks.

Table I summarised the patients’ demographics, clinical and operative data as well as their recovery patterns.

**Table I:** Demographics, Clinical and Operative Data of the Patients and their Recovery Patterns

Parameters	Case 1	Case 2
Age (years)	76	53
Gender	Female	Male
Past Medical History	HTN, BA	DM, CAD
Diagnosis	T11/12 stenosis	CSM
Index Surgery	PDF T11/12	Laminectomy & LMF C4-C6
**Spinal Epidural Haematoma**		
Presenting Symptoms	Increasing lower limb weakness that worsened on supine position	Severe neck pain, weakness of right upper limb and numbness both thighs
Onset of Symptoms	POD 4	POD 4
Risk Factors	Advanced age	Aspirin, DM
Surgery Performed	Evacuation of haematoma	Evacuation of haematoma
Time from Onset of Symptoms to Surgery (day)	4	1
Time taken for Recovery of Neurological Deficit to Pre-operative Status (week)	6	2

Abbreviations. HTN: hypertension, BA: bronchial asthma, DM: diabetes mellitus, CAD: coronary artery disease, CSM: cervical spondylotic myelopathy, PDF: posterior decompression and fusion, LMF: lateral mass fusion, POD: post-operative day.

## Discussion

SEH is commonly caused by bleeding from the epidural venous plexus. The thoracic spine was the most common area of involvement^3^. Aono *et al*, reviewed 6,356 patients who underwent decompression surgeries and reported 26 patients (0.41%) required SEH evacuation. PSEH occurred most commonly after thoracic laminectomy (4.46%) followed by posterior lumbar interbody fusion (0.67%), and lumbar laminectomy (0.5%)^2^.

Various aetiologies for SEH had been reported. These included vascular anomalies such as arteriovenous malformation, vertebral hemangiomas, vertebral fractures, birth trauma, lumbar punctures, epidural procedures, surgical bleeding, hypertension, diabetes mellitus, hepatitis C, bleeding disorders or anticoagulants usage^1,3^. In DPSEH, some authors suggested age, multilevel procedures, scar tissue due to previous spinal surgery and heparinisation were risk factors^3-5^. In our series, our second patient with diabetes and coronary artery disease was on aspirin. The aspirin was withheld five days prior to surgery as per guidelines. The optimal duration of discontinuation of antiplatelet therapy such as aspirin may need to be reexplored and investigated in future studies. In patient who are on antiplatelet or anticoagulant therapy, higher vigilance is needed. Additional measures such as delayed removal of drain(s), insertion of additional drain as a remedial to the possibility of blocked primary drain and delayed reinitiating antiplatelet or anticoagulant therapy post-operatively may need to be considered.

Previous studies had described that the duration of onset for DPSEH varied^1,3-5^. Surgeons should bear in mind that symptomatic DPSEH may occur up to two weeks from the index surgery^5^. Uribe *et al* reported seven DPSEH out of 4,018 patients with an average time to neurological deficit of 5.3 days^3^. Anno *et al* reported six patients had SEH after a delay of five days^1^. Sokolowski *et al* reported an even more delayed onset of 13 days from index surgery in four patients^4^. Recognising the initial clinical presentations of DPSEH is crucial. In Case 1, there was a sudden onset of new neurologic deterioration while in Case 2, there was severe neck pain associated with neurological symptoms. These findings were consistent with the report by Uribe *et al*, who reported severe sharp pain with radiation to the extremities as the initial presenting symptom^3^. It is imperative to acknowledge that a sudden onset of severe sharp pain as an initial symptom of SEH.

The neurological recovery in Case 1 was slower than Case 2 (Table I). One possible factor was the advanced age noted in Case 1. Elderly patients tend to have profound muscle wasting that could affect the neurological recovery. In addition, the delay in the surgical evacuation of haematoma in Case 1 could be a contributing factor to her slower recovery pattern.

This case series highlighted that surgeons should have high index of suspicion of delayed onset of SEH especially in patients with risk factors. Patients need to be informed during the consent taking of this rare but devastating complication. Timely surgical intervention should be undertaken to avoid irreversible neurological deficit.
